# Cell Cycle-Dependent Microtubule-Based Dynamic Transport of Cytoplasmic Dynein in Mammalian Cells

**DOI:** 10.1371/journal.pone.0007827

**Published:** 2009-11-13

**Authors:** Takuya Kobayashi, Takashi Murayama

**Affiliations:** Department of Pharmacology, Juntendo University School of Medicine, Tokyo, Japan; University of Minnesota, United States of America

## Abstract

**Background:**

Cytoplasmic dynein complex is a large multi-subunit microtubule (MT)-associated molecular motor involved in various cellular functions including organelle positioning, vesicle transport and cell division. However, regulatory mechanism of the cell-cycle dependent distribution of dynein has not fully been understood.

**Methodology/Principal Findings:**

Here we report live-cell imaging of cytoplasmic dynein in HeLa cells, by expressing multifunctional green fluorescent protein (mfGFP)-tagged 74-kDa intermediate chain (IC74). IC74-mfGFP was successfully incorporated into functional dynein complex. In interphase, dynein moved bi-directionally along with MTs, which might carry cargos such as transport vesicles. A substantial fraction of dynein moved toward cell periphery together with EB1, a member of MT plus end-tracking proteins (+TIPs), suggesting +TIPs-mediated transport of dynein. In late-interphase and prophase, dynein was localized at the centrosomes and the radial MT array. In prometaphase and metaphase, dynein was localized at spindle MTs where it frequently moved from spindle poles toward chromosomes or cell cortex. +TIPs may be involved in the transport of spindle dyneins. Possible kinetochore and cortical dyneins were also observed.

**Conclusions and Significance:**

These findings suggest that cytoplasmic dynein is transported to the site of action in preparation for the following cellular events, primarily by the MT-based transport. The MT-based transport may have greater advantage than simple diffusion of soluble dynein in rapid and efficient transport of the limited concentration of the protein.

## Introduction

Cytoplasmic dynein is a microtubule (MT) minus end-directed molecular motor and plays an important role in many cellular functions including organelle positioning, vesicle transport, and cell division [1]. Cytoplasmic dynein forms a huge multisubunit protein complex (∼1.5 MDa) composed of two identical heavy chains, two 74-kDa intermediate chains (IC74), four light intermediate chains (LICs), and up to three light chain dimers (LC8, TcTex-1, and Roadblock) [2], [3]. The heavy chain which is a member of the AAA ATPase protein superfamily carries a motor activity [4], and the other subunits comprising the cargo binding domain may play a regulatory role for intracellular transport [1].

In interphase cells, dynein transports vesicles, organelles, and other types of cargos from the cell periphery toward the cell center (to the minus end of MTs). This is opposite direction of transport by kinesin, a MT plus end-directed motor (from the cell center to the cell periphery). During mitosis, cytoplasmic dynein plays various roles, such as spindle organization, chromosome capture and congression, spindle assembly checkpoint (SAC) protein removal, and anaphase chromosome motility [5], [6], [7], [8], [9]. In accordance with these various roles, dynein is found at various sites, i.e., mitotic spindles, spindle poles, kinetochores, and cell cortex [10], [11], [12], [13]. Kinetochore dynein may be important in chromosome capture and congression and SAC protein removal. The polar and cortical locations of dynein are consistent with roles in spindle assembly and positioning.

Cytoplasmic dynein heavy chain is encoded by a single gene and is expressed stably and constitutively during the cell cycle [1]. This is in marked contrast to kinesins, which include a number of isoforms having different functions and are expressed in cell cycle-dependent manner [14]. To accomplish multiple functions, dynein interacts with various proteins which include dynactin [15], kinases [16], and endosomal proteins [17]. Dynactin is a large protein complex necessary for cargo binding of dynein and interact with various proteins, thus linking them with dynein [18], [19]. These interactive partners might affect not only binding of the transport cargo, but also motor activity of dynein. Interaction with specific partners must be critically important in cell cycle-dependent roles of dynein.

Although much information has accumulated about roles and regulations of cytoplasmic dynein as described above, it has not fully been understood about regulatory mechanism of cell cycle-dependent distribution of dynein in living cells. Direct visualization of dynein by live-cell imaging technique would greatly help understand this issue. It has recently been shown that IC74 is a useful probe for functional dynein complex in living cells [20], [21]. IC74 tightly binds heavy chains and provides binding sites for three light chains [22], [23], [24], [25], [26] and p150^Glued^, a subunit of dynactin [15], [27]. Thus, IC74 is an important ‘scaffold’ protein for dynein complex formation.

To know behavior of cytoplasmic dynein in living cells, we generated a stable HeLa cell line expressing green fluorescence protein (GFP)-tagged IC74. We demonstrate that GFP-tagged IC74 is incorporated into functional dynein complex and monitors a cell cycle-dependent dynamic behavior of cytoplasmic dynein. Our findings suggest that dynein is transported to the site of action in preparation for the following cellular events primarily by the MT-based transport.

## Results

### Characterization of HeLa cells stably expressing fluorescent IC74

**Figure 1:**
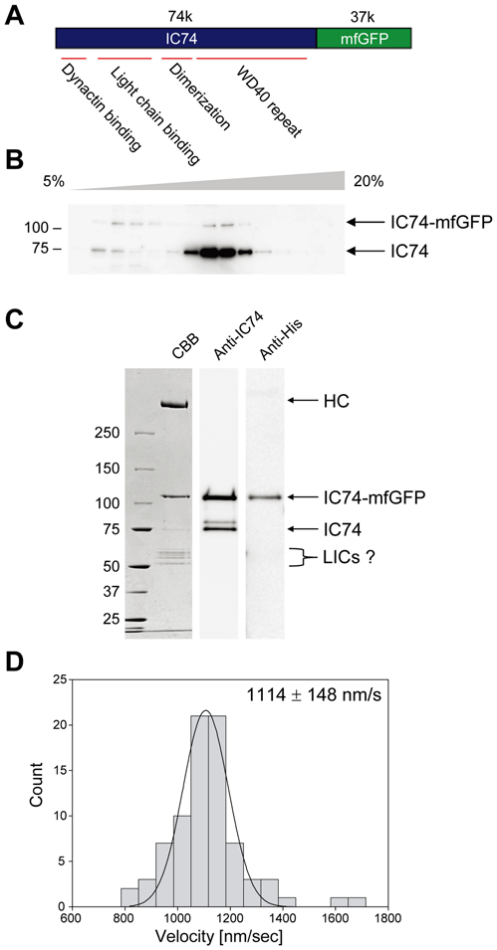
Incorporation of IC74-mfGFP into functional cytoplasmic dynein complex. **A.** Schematic diagram of IC74-mfGFP. mfGFP was fused at the C-terminus of IC74. **B.** Sucrose density gradient sedimentation of lysate from IC74-mfGFP HeLa cells. Lysate was sedimented into 5–20% sucrose gradient. Fractions were analyzed by Western blotting with antibody specific to IC74 (74.1). IC74-mfGFP was <5% of endogenous IC74. About half of IC74-mfGFP was sedimented to heavy sucrose fractions, indicating incorporation into dynein complex. **C.** SDS-PAGE and Western blotting of the purified IC74-mfGFP fraction. IC74-mfGFP was purified by StrepTrap column chromatography. The purified fraction contained a high molecular mass polypeptide (>250 kDa) probably of heavy chain (HC) in addition to IC74-mfGFP (∼110 kDa). Polypeptides at 50–60 kDa range could be LICs. Endogenous IC74 was also detected, indicating heterodimer formation of IC74-mfGFP and IC74. IC74-mfGFP was detected by antibody specific to tetrahistidine which reacts with 8×His tag in mfGFP. **D.** Histograms of velocity distribution of minus end-directed MTs in *in vitro* MT-gliding assay with the purified IC74-mfGFP fraction.

We generated a HeLa cell line stably expressing fluorescent IC74 using a fusion protein of IC74 and multifunctional GFP (mfGFP). mfGFP is a GFP mutant in which multiple affinity tags are inserted in tandem into an internal loop, and thus a useful tool both for live-cell imaging and biochemical characterization of protein complex [28]. mfGFP was fused at the C-terminus of IC74, near the WD40 repeat that is involved in binding to the heavy chain (**Fig. 1A**). HeLa cells expressing IC74-mfGFP showed no apparent abnormality in morphology and growth (data not shown). Sucrose density gradient sedimentation of the HeLa cell lysate demonstrated that the expressed IC74-mfGFP was <5% of endogenous IC74, and that about half of IC74-mfGFP was sedimented to heavy sucrose fractions, suggesting incorporation of IC74-mfGFP into dynein complex (**Fig. 1B**). Correspondingly, the purified IC74-mfGFP fraction by StrepTrap column chromatography contained a high molecular mass polypeptide (>250 kDa) probably of dynein heavy chain, in addition to IC74-mfGFP (∼110 kDa) (**Fig. 1C**). Several polypeptides were also seen at 50–60 kDa range which could be LICs. The purified fraction also contained endogenous IC74, suggesting heterodimer formation of IC74-mfGFP with endogenous IC74. In *in vitro* MT-gliding assay, the purified IC74-mfGFP fraction exhibited a minus end-directed motor activity with an averaged velocity of 1114±148 nm/sec (mean±SD, n = 80) (**Fig. 1D**). Taken together, these findings indicate that the expressed IC74-mfGFP is successfully incorporated into functional dynein complex.

### Behavior of cytoplasmic dynein in interphase cells

**Figure 2:**
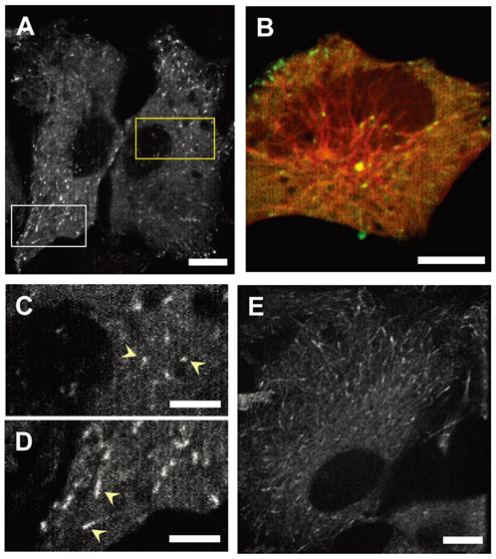
Live cell imaging of cytoplasmic dynein in IC74-mfGFP HeLa cells. **A.** Dynein is observed as moving fluorescent foci in the cytosol (see Movie S1). **B.** IC74-mfGFP HeLa cells were transfected with the mCherry-α-tubulin expression vector. Green and red represent dynein and MTs, respectively. Fluorescent foci are moving along with MTs (see Movie S2). **C, D.** Boxed areas in **A** are shown at higher magnification representing two classes of moving foci: spot-like foci (**C**, yellow box in **A**) and comet-like foci (**D**, white box in **A**). **E.** Distribution of dynein foci in methanol-fixed cells. The comet-like foci became clearly visible by disappearance of the spot-like foci. Scale bars: **A**, **B**, **D**, 10 µm; **D**, **E**, 5 µm.

Live-cell imaging of IC74-mfGFP HeLa cells was carried out by high-speed laser scanning confocal microscopy (see Materials and Methods). In interphase cell, IC74-mfGFP distributed as discrete foci which moved in the cytosol in addition to cytosolic nonstructural fluorescence (**Fig. 2A** and **Movie S1**). Labeling of MTs with mCherry-α-tubulin revealed that these foci moved along with MTs (**Fig. 2B** and **Movie S2**). We referred to the direction of moving foci as ‘centripetal’ and ‘centrifugal’ in which foci move toward the nucleus and the cell periphery, respectively. Based on their shape and behavior, we classified the discrete foci into two groups: ‘spot-like’ foci and ‘comet-like’ foci. The spot-like foci are round-shaped, seen throughout the cytosol, and exhibit rapid movement at both centripetal and centrifugal directions (**Fig. 2C**), whereas the comet-like foci are comet-shaped, seen mostly at the cell periphery, and slowly move centrifugally (**Fig. 2D**, see also **Movie S1**).

**Figure 3:**
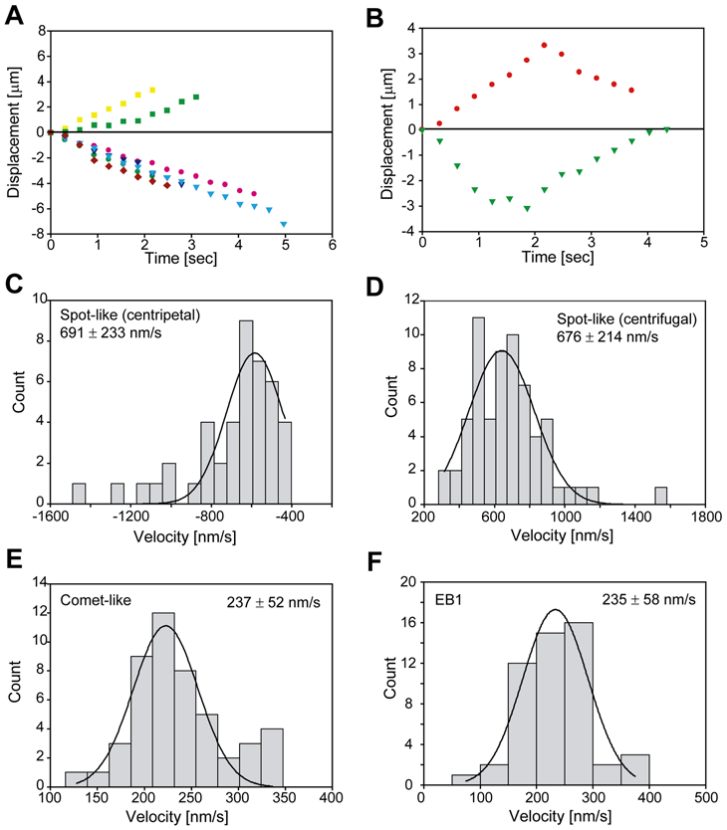
Movement analysis of fluorescent foci in interphase cells. **A.** Time-dependent displacements of six spot-like foci in the cytosol. Centrifugal movement (toward cell periphery) was recorded as positive and centripetal movement (toward nucleus) as negative displacement. **B.** Examples of dynein fluorescent foci which reversed the direction during observation. **C, D.** Histograms of velocity distributions of the spot-like foci toward the centripetal (**C**) and centrifugal (**D**) directions. The continuous lines correspond to a Gaussian fitting with the best-fitting velocity indicated in each figure. **E.** Histogram of velocity distribution of the comet-like foci. **F.** Histogram of velocity distribution of EB1-GFP in interphase HeLa cells.

The spot-like foci moved bi-directionally (**Fig. 3A**) and some foci reversed the direction during observation (**Fig. 3B**). Turning direction was frequently observed, probably due to crossing the intersecting MTs. In addition, some foci exhibited jiggling motion with no net motility (**Movie S1**). The velocity measurement by frame-to-frame tracking of the sequential images revealed that velocities of the spot-like foci at centripetal and centrifugal direction were 691±233 nm/sec (n = 43) and 676±214 nm/sec (n = 65), respectively (**Figs. 3C and 3D**). Some foci changed their velocity during movement (green squares in **Fig. 3A**). The properties of the spot-like foci are similar to those determined by fluorescent ligands for plasma membrane receptors that represent some endosomes [29], [30]. The spot-like foci might be a population of dynein which carries cargos such as transport vesicles.

The comet-like foci became clearly visible by methanol fixation of cells, which removes the spot-like foci (**Fig. 2E**). It completely disappeared by treatment of 10 µM paclitaxel that stabilizes MTs (data not shown). The velocity of the comet-like foci was 237±52 nm/sec (n = 48), which was about one third of the velocity of the spot-like foci (**Fig. 3E**). These properties are very similar to those of the MT plus end-tracking proteins (+TIPs) [31], [32]. Expression of the GFP-tagged EB1 in HeLa cells demonstrated similar behavior and velocity (235±58 nm/sec, n = 51) to the comet-like foci (**Figs. 3F** and **Movie S3**).

**Figure 4:**
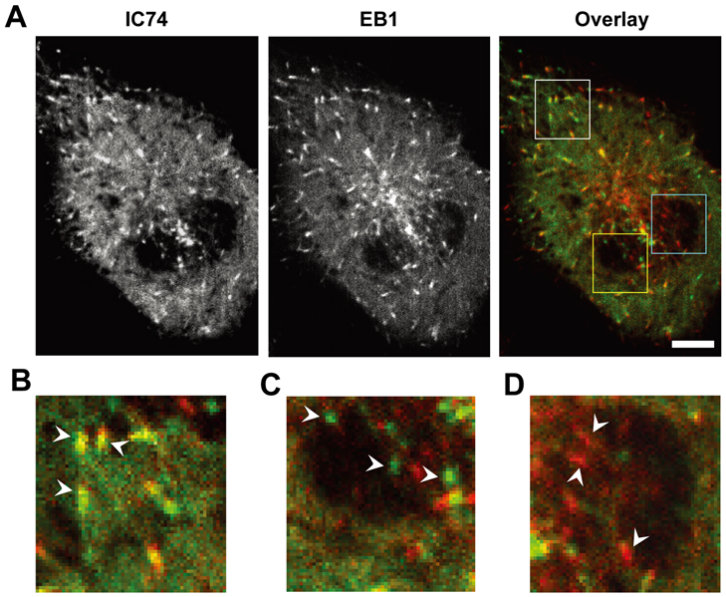
Simultaneous observations of IC74 and EB1 in interphase cells. **A.** EB1-mCherry was expressed in IC74-mfGFP HeLa cells and behaviors of IC74 and EB1 were simultaneously observed using W-view system. **B**, **C**, **D.** Boxed areas in **A** are shown at higher magnification. Colocalization of the comet-like and EB1 are clearly observed at cell periphery (**B**, white box in **A**). The spot-like foci were not colocalized with EB1 (**C**, yellow box in **A**). The comet-like foci are hardly seen near cell center where EB1 is present (**D**, cyan box in **A**). Because focal plane was set close to bottom of the cell, microtubule networks under the nucleus are seen which cross the nucleus. Scale bars: **A**, 10 µm; **B**, **C**, 5 µm.

To further investigate relationships between dynein and EB1, we observed these proteins simultaneously by multicolor imaging. Expression of mCherry-tagged EB1 in IC74-mfGFP HeLa cells revealed that the comet-like foci were colocalized with EB1 near the cell periphery (**Figs. 4A and 4B**, and **Movie S4**). In contrast, no colocalization was seen between the spot-like foci and EB1 (**Fig. 4C**). Interestingly, colocalization of IC74 and EB1 was clearly observed at the cell periphery but not near the cell center where the comet-like foci was hardly seen (**Fig. 4D**). Taken together, these findings suggest that cytoplasmic dynein may be transported to the cell periphery by interacting with +TIPs in interphase cells.

### Behavior of cytoplasmic dynein during mitosis

**Figure 5:**
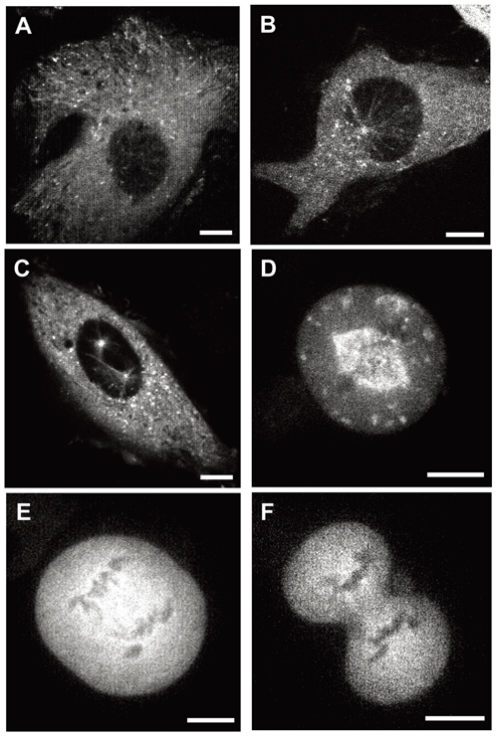
Behavior of cytoplasmic dynein in mitosis. **A.** Interphase. Dynein is distributed in the cytosol as the discrete foci. No fluorescence is detected in the centrosome and MTs. **B.** Late-interphase. Dynein is localized at the centrosome and radial MT array in addition to the moving foci. **C.** Prophase. Localization of dynein in the daughter centrosomes and radial MT arrays are remarkable. **D.** Metaphase. Spindle MTs are heavily stained by dynein fluorescence. Island-like foci are also observed near the cell cortex, which might be cortical dynein. **E.** Telophase. Dynein fluorescence increased in the cytosol with decrease on the spindle MTs. **F.** Cytokinesis. No apparent dynein fluorescence is detected on the spindle MTs nor mid body. Scale bars: **A–F**, 10 µm.

We next determined behavior changes of cytoplasmic dynein during mitosis. IC74-mfGFP HeLa cells were synchronized at the G1/S boundary using double thymidine treatment. In late-interphase cells, the centrosome and the radial MT array became clearly visible by dynein fluorescence (**Fig. 5B** and **Movie S5**). This is in marked contrast to interphase cells in which the centrosome and the radial MT array were undetectable (**Fig. 5A**). This difference is unlikely to be due to changes in cell shape and centrosome position, because the centrosome and the radial MT array can be clearly seen by tubulin staining (see **Fig. 2B**). Dynein fluorescence on the radial MT array appeared to be relatively homogeneous and immotile. In addition, the spot-like foci moved on the radial MT array (**Movie S5**). Thus, dynein fluorescence on the radial MT array might be different from the moving foci. In late-interphase cells, centripetal movement of the spot-like foci appeared to be more frequent than in interphase. In prophase when the daughter centrosomes start to separate, dynein fluorescence on the daughter centrosomes and the radial MT array became more remarkable (**Fig. 5C**). The spot-like foci moved along with MTs as in interphase and late-interphase, whereas the comet-like foci were rarely detectable in prophase.

**Figure 6:**
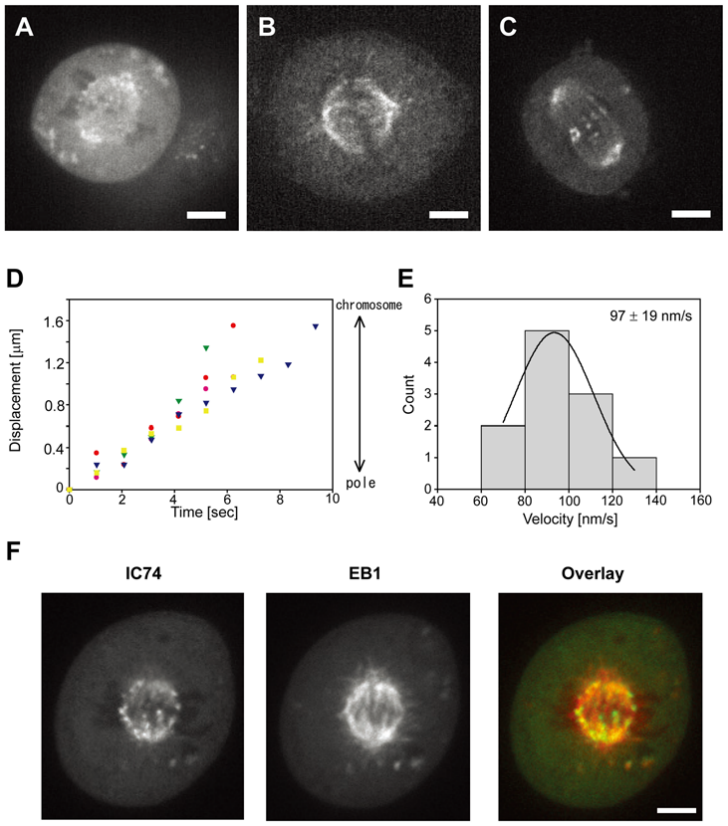
Behavior of dynein in prometaphase and metaphase. **A.** Many fluorescent foci are moving on the spindle MTs in prometaphase. **B.** Methanol-fixed cell. These fluorescent foci are resistant to methanol fixation. **C.** Treatment with paclitaxel markedly reduced the moving foci, and visualized dynein at spindle poles and close to chromosomes (possibly kinetochores). **D.** Time-dependent displacements of the fluorescent foci on the spindles MTs. Movement from spindle poles toward chromosomes is recorded as positive displacement. **E.** Histogram of velocity distributions of the fluorescent foci on the spindle MTs. **F.** Simultaneous observations of IC74 and EB1 at the mitotic spindles in prometaphase. mCherry-EB1 was expressed in IC74-mfGFP HeLa cells. Fluorescent foci at the spindle MTs are colocalized with EB1. Some foci near chromosomes exhibit no colocalization with EB1, which might be kinetochore dynein.

In prometaphase and metaphase cells, dynein was localized at spindle MTs and spindle poles (**Figs. 5D** and **6A**, and **Movie S6**). This fluorescence was primarily due to a number of fluorescent foci moving from spindle poles toward chromosomes. The velocity of moving foci was 97±19 nm/sec 2 (n = 11) (**Figs. 6D and 6E**). The fluorescence remained unchanged after fixation with methanol (**Fig. 6B**). Treatment of cells with 10 µM paclitaxel caused a dramatic reduction in the number of moving foci (**Fig. 6C** and **Movie S7**). Correspondingly, the moving foci were colocalized with EB1 (**Fig. 6F** and **Movie S8**). These findings suggest that the moving foci on the spindle MTs may represent +TIPs-mediated transport of dynein. We also observed that some foci are moving on the spindle MTs at both directions even in the presence of paclitaxel (**Movie S7**). This suggests the presence of some motor-driven transports by kinesins or dynein on the spindle MTs.

In addition to the moving foci, we observed some foci staying close to the chromosomes which are most prominent in prometaphase (**Movies S6** and **S8**). These foci were still visible after paclitaxel treatment (**Fig. 6C** and **Movie S7**) and not colocalized with EB1 (**Fig. 6F** and **Movie S8**), suggesting no interactions with +TIPs. They might be kinetochore dynein [10], [33]. Dynein was also localized as several ‘island-like’ foci near the cortical sites (**Figs. 5D** and **6A**). Live-cell imaging at higher magnification revealed that these island-like foci are clusters of small distinct foci which seem to be weakly connected to each other (**Movie S6**). They were localized primarily parallel to the equatorial plane in prometaphase (**Fig. 6A**) and more widely distributed along with cortex in metaphase (**Fig. 5D**). These properties seem to be consistent with those of cortical dynein [12]. We found that a number of foci were moving from the spindle poles toward the cortical sites (**Movie S6**), suggesting possible transport of dynein along with the astral MTs to the cortex.

In anaphase to telophase, dynein fluorescence increased in the cytosol (**Fig. 5E**). The moving foci were rarely observed on the spindle MTs. In the end of cytokinesis, dynein fluorescence and moving foci were hardly seen in the spindle MTs (**Fig. 5F**). No dynein fluorescence was detected at the mid body.

### Behavior of cytoplasmic dynein visualized by fluorescent LIC1

The important issue using IC74-mfGFP as a functional probe for cytoplasmic dynein is whether the moving foci represent dynein complex containing the heavy chain. The fact that a part of the expressed IC74-mfGFP is not incorporated into dynein complex (see **Fig. 1B**) would raise a possibility that some or all of the moving foci might represent IC74 lacking dynein heavy chain. To check this possibility, we tried to visualize dynein by fluorescent LIC which is an essential component of dynein complex [1]. Recent study has demonstrated that IC74 and LIC bind to the heavy chain but not to each other [34]. Therefore, colocalization of IC74 and LIC would strongly support the presence of the heavy chain.

**Figure 7:**
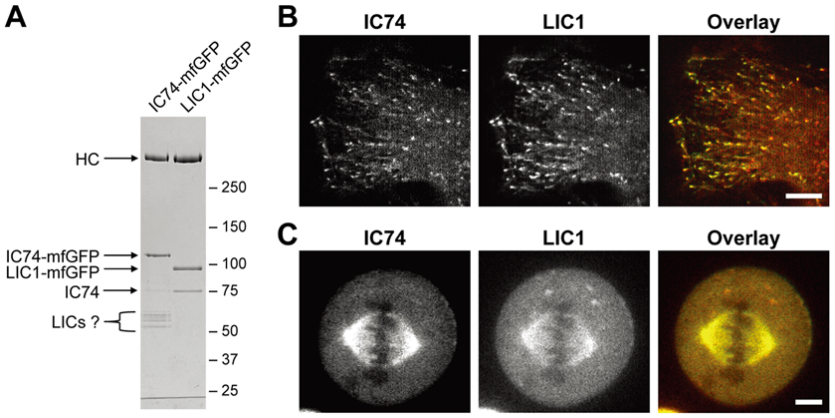
Fluorescent LIC1 as a probe for cytoplasmic dynein. **A.** LIC1-mfGFP was purified from lysate of the LIC1-mfGFP expressing cells by StrepTrap chromatography and the protein composition was compared with that of the purified IC74-mfGFP fraction. Both heavy chain and IC74 were co-purified with LIC1-mfGFP. Polypeptides at 50–60 kDa range seen in the IC74-mfGFP fraction are undetectable in the purified LIC1-mfGFP fraction. **B**, **C.** IC74-mfGFP HeLa cells were transfected with LIC1-mCherry. Both the spot-like and the comet-like foci were labeled by LIC1-mCherry in interphase cells (**B**). Colocalization of IC74-mfGFP and LIC1-mCherry is also observed at mitotic spindle in metaphase cells (**C**). Scale bars: **B**, **C**, 10 µm.

We made expression constructs of human LIC1 which was tagged with mfGFP or mCherry at the C-terminus. The purified LIC1-mfGFP fraction from the HEK cell lysate contained three bands: a high molecular mass polypeptide (>250 kDa) probably of the heavy chain, LIC1-mfGFP (∼90 kDa), and IC74 (∼75 kDa) (**Fig. 7A** **, right**). Thus, LIC1-mfGFP is successfully incorporated into dynein complex, as true of IC74-mfGFP (**Fig. 7A** **, left**). The LIC1-mfGFP fraction did not contain 50–60 kDa polypeptides seen in the IC74-mfGFP fraction. This seems to be consistent with an idea that these polypeptides are endogenous LICs which can be displaced by LIC1-mfGFP.

Localization and behavior of LIC1 was examined by transiently expressing LIC1-mCherry in IC74-mfGFP HeLa cells. In interphase cells, LIC1 exhibited good colocalization with IC74 in both the spot-like and the comet-like foci (**Fig. 7B** and **Movie S9**). Colocalization was also confirmed at the moving foci on mitotic spindle MTs (**Fig. 7C**). These findings strongly support that the moving foci both in interphase and during mitosis probably represent dynein complex containing the heavy chain.

## Discussion

**Figure 8:**
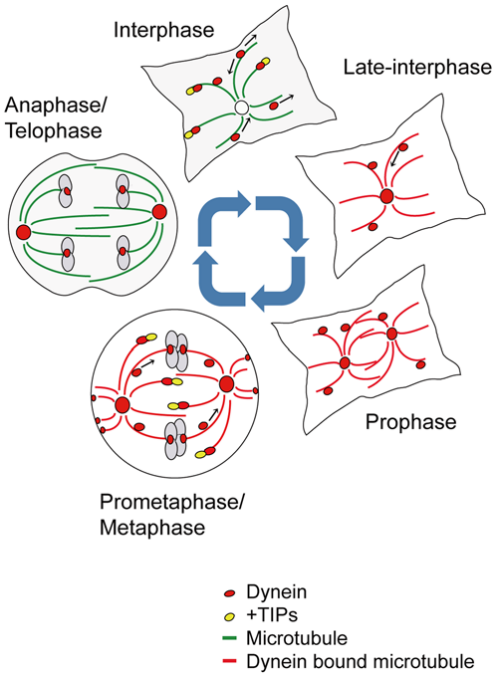
Schematic drawing of cell cycle-dependent dynamic behavior of cytoplasmic dynein in HeLa cells. In interphase, dynein may be associated with cargos which move bi-directionally (as the spot-like foci). A substantial population of dynein is transported toward cell periphery by +TIPs (as the comet-like foci). Dynein is not associated with the centrosome and MTs. In late-interphase, dynein is localized at the centrosome and the radial MT array. Centripetal movement of possible cargo-associated dynein is increased. In prophase, association of dynein with the daughter centrosomes and radial MT arrays are remarkable. In metaphase, dynein is associated with spindle poles and spindle MTs and is frequently transported toward chromosomes, probably by +TIPs. The motor-driven transport may also be present. In addition, dynein may be localized near chromosomes as kinetochore dynein and near cortex as cortical dynein. In telophase, spindle-associated dynein decreases, whereas cytosolic dynein increases.

In this study, we have visualized cytoplasmic dynein with mfGFP-tagged IC74 in living HeLa cells. We found that behavior of cytoplasmic dynein dramatically changes in a cell-cycle dependent manner (**Fig. 8**). In interphase, dynein distributes as spot-like foci which might carry the cargos or as comet-like foci which are colocalized with EB1 on the growing MTs. In late-interphase, dynein is concentrated in the centrosome and the radial MT array. In prophase, localization at the daughter centrosomes and radial MT arrays is remarkable. In prometaphase to metaphase, dynein is localized at spindle MTs where it frequently moves from spindle poles toward chromosomes and cortex. Possible kinetochore and cortical dyneins are also seen. In anaphase to telophase, spindle-associated dynein decreases, whereas cytosolic dynein increases. These findings correspond to the previous reports of localization of dynein by immunostaining of dynein subunits with fixed cells [10], [11], [12], [33], [35], [36], [37].

### GFP-tagged IC74 as a functional probe for cytoplasmic dynein

Use of IC74 as a functional probe for cytoplasmic dynein has initially been demonstrated with PC12 cells, in which all the stably expressed GFP-tagged IC74 is successfully incorporated into dynein complex [20]. A recent report, however, has claimed no incorporation of the transiently expressed GFP-tagged IC74 in dynein complex in COS-7 cells [38]. In the present study, we demonstrate that about half of the stably expressed IC74-mfGFP is incorporated into functional dynein complex. Because GFP tag is fused at the C-terminus of IC74 in all three studies, position of the GFP tag seems unlikely to be the reason for the difference. Cell type (PC12 and HeLa vs. COS-7) or expression system (stable expression vs. transient expression) could be possible cause.

In our IC74-mfGFP HeLa cells, about half of IC74-mfGFP is not incorporated into dynein complex. One might assume that such unincorporated IC74-mfGFP affects the fluorescence measurements in live-cell imaging. Nevertheless, we believe that the moving foci in interphase (spot-like and comet-like foci) and those during mitosis (on the spindle MTs) represent dynein complex containing the heavy chain, because these foci were also labeled by LIC1, a component of dynein complex which is reported to bind to the heavy chain independently of IC74 [34]. Although cellular distribution of the unincorporated IC74-mfGFP remains unclear, it could partly be responsible for cytosolic nonstructural fluorescence.

### +TIPs-mediated transport of cytoplasmic dynein in interphase

+TIPs are highly diverse group of the MT-associated proteins and thought to be involved in regulation of dynamic properties of MTs [31], [32]. The MT plus-end accumulation of cytoplasmic dynein has been visualized in the fungus *Aspergillus nidulans* using GFP-tagged heavy chain *nudA* [39]. In mammalian cells, however, no live-cell imaging of dynein at the MT plus ends has so far been reported, although colocalization of endogenous IC74 with CLIP-170 was shown in fixed COS-7 cells [35]. We here clearly demonstrated that cytoplasmic dynein complex is moving on MTs toward the cell periphery together with EB1. This supports the hypothesis of +TIPs-mediated transport of cytoplasmic dynein [35]. Interestingly, the comet-like foci of dynein were hardly seen near cell center where EB1 is present. Interaction of dynein and +TIPs is thought to be mediated by dynactin [35], [40]. It is possible that dynein-dynactin and/or dynactin-+TIPs interactions may be temporally and spatially regulated. Visualization of dynactin or other +TIPs components would address the underlying mechanism of this regulation.

### Cytoplasmic dynein in late-interphase and prophase

Cytoplasmic dynein is localized at centrosome in late-interphase. This localization is more profound in prophase. Cell cycle-dependent centrosome localization of dynein is consistent with the previous report by immunostaining of IC74 in COS-7 cells [37]. Dynactin is localized at centrosomes throughout the cell cycle [37]. Thus, centrosome localization of dynein might be regulated via interactions of dynein with dynactin and centrosomal components. In late-interphase and prophase, dynein fluorescence was also observed on the radial MT array which seems to be relatively homogeneous and immotile. This might suggest direct or indirect interaction of dynein with MTs. Because such localization does not occur in interphase, some cycle-dependent changes might be involved in the interaction of dynein with MTs.

### Cytoplasmic dynein in prometaphase and metaphase

We have demonstrated that cytoplasmic dynein is localized at three different sites in prometaphase and metaphase: on the spindle MTs, at kinetochores, and near cortex. These findings are well consistent with immunohistochemical localization of dynein with fixed cells: spindle dynein [10], [11], kinetochore dynein [33], [36], and cortical dynein [12]. Several novel findings have been obtained by live-cell imaging of these dyneins. On spindle MTs, majority of dynein is transported by +TIPs on the growing MTs. +TIPs-mediated transport of dynein also occurs on the astral MTs. Thus, +TIPs-mediated transport of dynein may be important for supply of dynein during mitosis. Kinetochore dynein is thought to be involved in attachment, orientation, and alignment of kinetochores to MTs [41]. We observed that kinetochore dynein is resistant to paclitaxel treatment and not colocalized with EB1, suggesting that kinetochore dynein may not be interacted with +TIPs. An interesting question is how kinetochore dynein is transported to the kinetochores. Cortical dynein is thought to be involved in proper spindle positioning during mitosis [42]. We found that several ‘island-like’ foci are localized near the cortical sites, which are linked with spindle poles by astral MTs, suggesting that they may be cortical dynein. Cortical dynein has been demonstrated by immunocytochemistry in MDCK cells, which is localized as spots at the cortex during late prometaphase and metaphase [12]. The island-like foci in our study, however, seem to be larger in size than the spots of cortical dynein observed with MDCK cells [12]. Difference in cell type could be one of possible reasons, because MDCK is a highly polarized cell line.

### Dynamic behavior of cytoplasmic dynein in cell cycle

Based on the findings in the present study, we would like to propose that cytosolic dynein may be effectively transported to the site of action in preparation for the following cellular events. In interphase, dynein is transported to the cell periphery by +TIPs. This seems to be supply for the minus end-directed transport of various cargos. Dyneins at the centrosome and radial MT array in late-interphase and prophase may be prepared for formation of spindle poles and spindle MTs in prometaphase and metaphase. Dynein at the spindle poles, in turn, may be used for supply for the kinetochore and the cortical dyneins. It should be noted that dynein is transported primarily by the MT-based transport systems, i.e., +TIPs (as comet-like foci in interphase and moving foci on the spindle MTs) and MT binding (on the radial MT array in late-interphase). The distribution of dynein by MT-based transport may have greater advantage than simple diffusion of soluble dynein in rapid and efficient transport of the limited concentration of the protein. It should also be noted that use of +TIPs and MT binding may be ‘cost effective’ compared with motor-driven transport in which no additional energies are required. Elucidation of the regulatory mechanisms of dynein transport will greatly help understand the MT-based intracellular transport system.

## Materials and Methods

### Generation of cDNA constructs

cDNA encoding 74-kDa dynein intermediate chain (IC74) 1B isoform was amplified from HEK cells by RT-PCR using a pair of primers: 5′-GGGGAGCTCATGTCTGACAAAAGTGACTTAAAA-3′ and 5′-GGGGGTACCGGCAGATAACTCAACAGT-3′, and subcloned into pIREShyg2 vector. mfGFP [28] was fused to the C terminus of IC74. cDNAs encoding α-tubulin, EB1, and LIC1 were amplified from HEK cells and subcloned into pcDNA5/FRT/TO vector. mCherry [43] was fused to either N-terminus (α-tubulin) or C terminus (EB1 and LIC1).

### Cell culture and transfection

HeLa cells were cultured in Dulbecco's Modified Eagle Medium (Invitrogen) supplemented with 10% fetal calf serum and 2 mM L-glutamine. For generation of stable transfectants of IC74-mfGFP, the expression vector was transfected to HeLa cells using Lipofectamine LTX reagent (Invitrogen). The transfected cells were screened by hygromycin (400 µg/ml) for 2–3 weeks. Hygromycin-resistant colonies were further screened by fluorescence microscopy and GFP-positive colonies were selected. Transient expression of mCherry-tagged proteins was carried out using Lipofectamine LTX and assayed 24 h after transfection.

### Live cell imaging

Live cell imaging was carried out with a high-speed confocal laser scanning microscope system. HeLa cells were grown on collagen-coated glass bottom dishes. Before observation, culture medium was changed to Krebs-Ringer Hepes (KRH) solution containing 140 mM NaCl, 3.6 mM KCl, 0.5 mM NaH_2_PO_4_, 2 mM NaHCO_3_, 0.5 mM MgSO_4_, 1 mM CaCl_2_, 10 mM glucose, and 10 mM Hepes, pH 7.4. The dish was placed on the stage of an inverted microscope with a Nipkow disc confocal laser scanning unit (CSU22, Yokogawa, Japan) equipped with an Argon Krypton Ion Laser (488 and 568 nm excitation). Cells were observed with a 100× objective lens (Plan Apo, N.A. = 1.40, Nikon, Japan) at room temperature, and images were acquired with an EM-CCD camera (C9100, Hamamatsu Photonics, Japan) at a rate of 310 msec/image (for interphase cells) or 1570 msec/image (for mitotic cells). The images were analyzed by Aquacosmos software (Hamamatsu Photonics, Japan). For movement analysis, the positions of the moving foci were determined by a two-dimensional Gaussian fitting algorithm with a custom program ‘Mark2’ [44]. The velocity of each foci was measured from displacements of >5 successive frames by a least-squares curve-fitting procedure.

For paclitaxel treatment, cells were incubated for 10 min in KRH solution containing 10 µM paclitaxel. For methanol fixation, cells were washed twice with KRH solution, incubated for 15 min in ice-cold methanol, and rinsed with PBS before observations. Double thymidine treatment for synchronization of the HeLa cells at the G1/S boundary was carried out as described [45]. For multicolor observations, cells were observed using W-view system (Model 8509, Hamamatsu Photonics, Japan).

### Sucrose density gradient sedimentation

Sucrose density gradient sedimentation of dynein complex was carried out as described [20] with some modifications. Briefly, lysate from IC74-mfGFP HeLa cells was prepared by homogenizing in a buffer A (0.1 M NaCl, 25 mM Tris-HCl, pH 7.5, 1 mM MgCl2, 0.1 mM ATP, 0.5 mM dithiothreitol) containing 0.05% Triton X-100 and complete mini protease inhibitor cocktail (Roche). After centrifugation at 100,000×g for 30 min, the supernatant was applied onto top of a 5–20% sucrose gradient in buffer A. The gradient was ultracentrifuged for 24 h at 100,000×g. The resultant fractions of the gradient were processed to SDS-PAGE and Western blotting for detection of IC74.

### Purification of dynein complex with IC74-mfGFP

Dynein complex was purified with SBP-tag in mfGFP [28]. Briefly, HeLa cells expressing IC74-mfGFP (ten 150 mm culture dishes) were collected and rinsed twice with phosphate buffered saline. The cell lysate was prepared by homogenizing the cells in buffer B (0.2 M NaCl, 50 mM Tris-HCl, pH 7.5, 10% sucrose, 5 mM MgCl2, 0.1 mM ATP, 0.5 mM dithiothreitol) containing 0.05% Triton X-100 and complete mini protease inhibitor cocktail (Roche). After centrifugation and filtration, the lysate was applied onto a StrepTrap HP column (GE healthcare) that had been equilibrated with buffer B. After washing with buffer A, bound proteins were eluted with buffer B containing 2.5 mM desthiobiotin.

### SDS-PAGE and Western blotting

Proteins were separated by SDS-PAGE using standard Laemmli's buffer system with 3–12% gradient gels and stained with Coomassie Brilliant Blue. For Western blotting, separated proteins were transferred onto PVDF membranes. Monoclonal antibodies against IC74 (74.1, Abcam, ab23905) and tetrahistidine (Qiagen, 34670) were used at 1∶1,000 and 1∶2,000 dilutions, respectively. Positive bands were detected by chemiluminescence using HRP-labeled anti-mouse IgG (KPL).

### In vitro MT gliding assay

The MT gliding on the purified dynein-coated coverslips was observed under a dark-field microscope as described [44].

## Supporting Information

Movie S1 Cytoplasmic dynein in interphase. Many moving foci are observed. These foci are classified into two groups: spot-like foci and comet-like foci.
(3.35 MB MOV)

Movie S2 Simultaneous observations of cytoplasmic dynein (green) and microtubules (red) in interphase. The discrete foci are moving along with the MTs.
(1.66 MB MOV)

Movie S3 Behavior of dynein and EB1 at the cell periphery. Left, IC74-mfGFP; Right, EB1-GFP.
(1.79 MB MOV)

Movie S4 Simultaneous observations of IC74-mfGFP (left, green in right) and EB1-mCherry (middle, red in right). The comet-like foci are colocalized wiht EB1, whereas the spot-like foci are not. The comet-like foci are hardly seen near the cell center, where EB1 is clearly observed.
(5.19 MB MOV)

Movie S5 Centrosome and radial MT array localization of cytoplasmic dynein in late interphase cells.
(3.41 MB MOV)

Movie S6 Cytoplasmic dynein in prometaphase. Many fluorescent foci are moving from the spindle poles toward chromosomes. Possible astral MTs are also seen on which dynein is moving toward to the cortex. Island-like foci close to the cell cortex might be cortical dyneins. Note that fluorescent foci near the center which move back and forth might be kinetochore dynein.
(0.74 MB MOV)

Movie S7 Cytoplasmic dynein in metaphase after treatment with paclitaxel. Paclitaxel (10 µM) caused a dramatic reduction in the spindle-associated dynein, and visualized dyneins at the spindle poles and kinetochores.
(1.10 MB MOV)

Movie S8 Simultaneous observations of IC74-mfGFP (green) and EB1-mCherry (red) at mitotic spindles in prometaphase. Moving foci on the spindle MTs are colocalized with EB1, whereas foci staying near chromosomes (possible kinetochore dynein) is not.
(0.37 MB MOV)

Movie S9 Simultaneous observations of IC74-mfGFP (left, green in right) and LIC1-mCherry (middle, red in right) in interphase cells. Both the spot-like and comet-like foci are colocalized with LIC1.
(5.47 MB MOV)
